# The Human LL-37(17-29) antimicrobial peptide reveals a functional supramolecular structure

**DOI:** 10.1038/s41467-020-17736-x

**Published:** 2020-08-04

**Authors:** Yizhaq Engelberg, Meytal Landau

**Affiliations:** 1 Department of Biology, Technion-Israel Institute of Technology, 3200003 Haifa, Israel; 2 Centre for Structural Systems Biology (CSSB), and European Molecular Biology Laboratory (EMBL), 22607 Hamburg, Germany

**Keywords:** Biochemistry, Biophysics, Antimicrobials, X-ray crystallography

## Abstract

Here, we demonstrate the self-assembly of the antimicrobial human LL-37 active core (residues 17–29) into a protein fibril of densely packed helices. The surface of the fibril encompasses alternating hydrophobic and positively charged zigzagged belts, which likely underlie interactions with and subsequent disruption of negatively charged lipid bilayers, such as bacterial membranes. LL-37_17–29_ correspondingly forms wide, ribbon-like, thermostable fibrils in solution, which co-localize with bacterial cells. Structure-guided mutagenesis analyses supports the role of self-assembly in antibacterial activity. LL-37_17–29_ resembles, in sequence and in the ability to form amphipathic helical fibrils, the bacterial cytotoxic PSMα3 peptide that assembles into cross-α amyloid fibrils. This argues helical, self-assembling, basic building blocks across kingdoms of life and points to potential structural mimicry mechanisms. The findings expose a protein fibril which performs a biological activity, and offer a scaffold for functional and durable biomaterials for a wide range of medical and technological applications.

## Abstract (web summary)

The human antibacterial and immunomodulatory peptide LL-37 is a hCAP-18 protein cleavage product that self-assembles. Here, the authors present the human and gorilla LL-37 (17–29) crystal structures, revealing a self-assembly of amphipathic helices into a densely packed and elongated hexameric structure with a central pore and mutagenesis experiments support the role of self-assembly for antibacterial activity.

## Introduction

The assembly of basic biological molecules into filamentous structures provides ample opportunities to design bioinspired materials for medical and technological applications^1–8^. One such application is addressing the urgent need to fight microbial aggressive, resistance, infections using materials which allow oral bioavailability, stability in harsh conditions, and long shelf-life. Antimicrobial peptides (AMPs) are canonical components of the innate immune system of many organisms^9^. AMP self-assembly bears functional relevance and can enhance antimicrobial activity^10^. Certain AMPs assemble into well-ordered fibrils that resemble amyloids^11–14^, which are proteins known to form cross-β fibrils composed of tightly mated β-sheets, and have been associated with neurodegenerative and systemic diseases^15,16^. Correspondingly, recent evidence of antimicrobial properties among some human amyloids suggests a potential physiological role of proteins otherwise known as pathological^17–21^.

Our previous findings demonstrated cross-α amyloid fibrillation of the cytotoxic phenol-soluble modulin α3 (PSMα3) peptide secreted by the pathogenic bacterium *Staphylococcus aureus*^22,23^. These fibrils are composed entirely of α-helices that stack perpendicular to the fibril axis into mated “sheets”, just as the β-strands assemble in amyloid cross-β fibrils^24^. PSMα3 is toxic to human cells, and some of its truncations and mutants show antibacterial activity^25–27^. Overall, PSMα3 provides a link between toxic activities against human and bacterial cells and unique helical amyloid fibrils^22,23^. Moreover, this architecture offers a scaffold for the design of various supramolecular nanostructures^1,8^. Although originating from different organisms, PSMα3 display sequence similarity with human LL-37 (hLL-37) (Supplementary Fig. 1), a hCAP-18 protein cleavage product which plays an important role in the first line of defense against pathogens^28^, which is also known to self-assemble^29,30^. Fibrillation of LL-37 was found critical for binding DNA and affecting receptors in the immune system^31^. Both PSMα3 and hLL-37 are cleaved in vivo into active truncations with diverse activities^32–36^. Some hLL-37 fragments show a diverse array of selectivity against microbial strains, and additional functions within the immune system^34,37,38^. Bacterial proteases can too cleave LL-37, supposedly for degradation or to release virulence factors^36^. Here we demonstrate that the active core peptide of hLL-37, containing residues 17–19 (hLL-37_17–29_), formed supra-helical highly stable fibrils that interact with bacterial cells. Structure-guided mutagenesis supported a plausible active fibril arrangement.

## Results

### The antibacterial LL37_17–29_ forms supra-helical fibrils

**Fig. 1:**
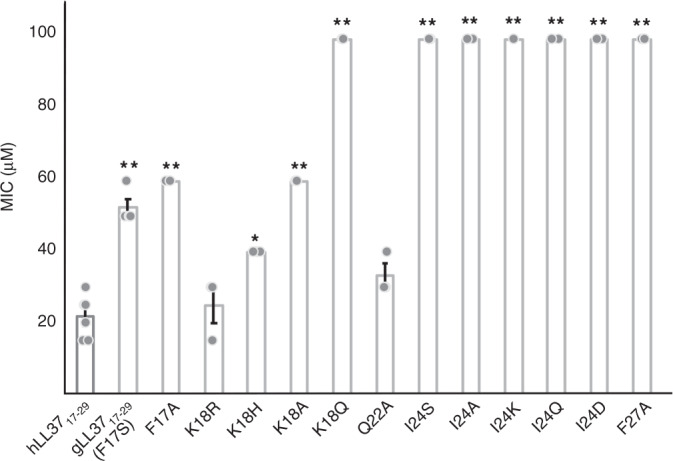
The effect of LL-37_17-29_ and its mutants on the growth of *M. luteus*. hLL-37_17-29_ and single-point mutants were incubated with *M. luteus* for 24h at a range of concentrations up to 100 µM, and bacterial growth rate was measured by optical density. From the resulting growth curves, the area under the curve (AUC) was calculated. MIC values were defined as the minimal concentration of the peptide which yielded less than 20% of the AUC of the control (bacteria with no added peptides). The mean MIC value of hLL-37_17-29_ is 22 µM. Mean MIC values of the F17A and K18A mutants, substituting residues lining the central pore, are 60 µM. Similarly, the mean MIC value gLL-37_17-29_ (equivalent to an F17S mutation) is 53 µM. The K18R, K18H and K18Q mutants showed mean MIC values of 25, 40, and >100 µM, respectively. The Q22A mutation, substituting a residue showing minimal contacts with other residues within the fibrillar assembly, showed a mean MIC value of 33 µM. The I24A, I24S, I24K, I24D and I24Q mutations, substituting a residue fully buried in the four-helix bundle, showed a mean MIC value of >100 µM. The F27A mutation, substituting a buried residue contacting other residues within the four-helix bundle and on surrounding helices in the fibrillar assembly, showed a mean MIC value of >100 µM. The experiments were performed at least three times, each on different days. Dots display measured values of individual repeats. Error bars indicate the standard error calculated between all measures. A *t*-test for two-sample assuming equal variances was performed, asterisk indicates *p* < 5 × 10^−03^ and double asterisk indicates *p* < 5 × 10^−05^, compared to hLL-37_17-29_. Source data are provided as a Source data file.

The hLL-37_17–29_ fragment was suggested to serve as the active core of the AMP, showing a different spectrum of antibacterial activity as compared to the full-length protein and other fragments, including being the shortest LL-37 fragment retaining anti-viral activity^36,39–41^. Although not directly detected in vivo, hLL-37_17–29_ can be cleaved from hCAP-18 or LL-37 by either proteinase K or staphylococcal peptidase I on its N-terminal side, and by trypsin on its C-terminal side. hLL-37_17–29_ is also the region within hLL-37 showing the highest sequence similarity to PSMα3 (Supplementary Fig. 1), and like the latter, forms a helical monomeric structure shown by NMR experiments^42^. More specifically, hLL-37_17–29_ generates an amphipathic helix with a larger hydrophobic moment compared to the entire hLL-37 and to PSMα3 (Supplementary Table 1). hLL-37_17–29_ elicited dose-dependent inhibition of Gram-positive *Micrococcus luteus* growth (Supplementary Fig. 2), with a minimal inhibitory concentration (MIC)^43^ of 22 µM (Fig. 1). It was also active against the *Staphylococcus hominis* bacterium, with a MIC of 39 µM (Supplementary Fig. 3).

**Fig. 2:**
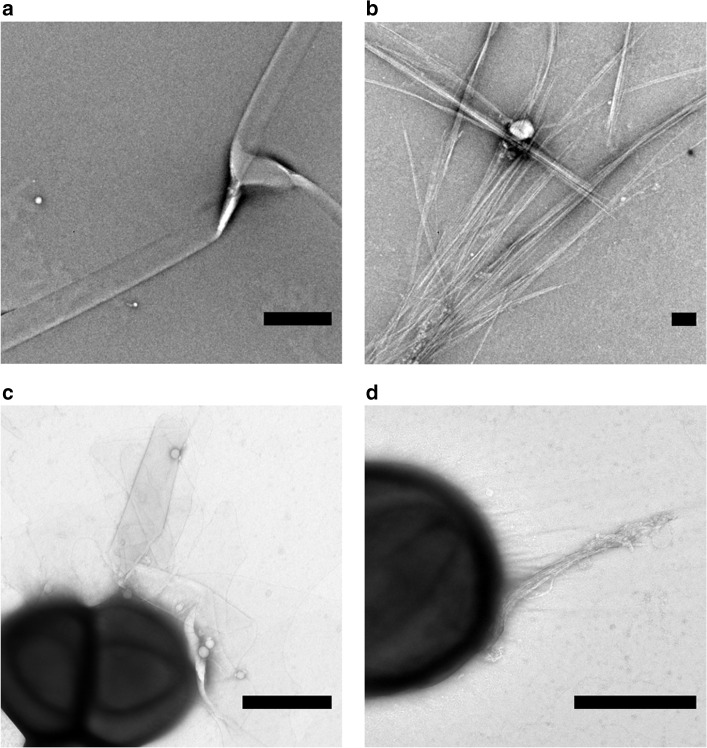
Human and gorilla LL-37_17-29_ fibrillar assemblies and interactions with bacteria. **a** An electron micrograph of 1 mM hLL-37_17-29_ incubated for three days. **b** An electron micrograph of 1 mM gLL-37_17-29_ incubated for 3 days. **c** An electron micrograph of 30 µM hLL-37_17-29_ (close to the MIC concentration of 22 µM) incubated with *M. luteus* for 4 h. **d** An electron micrograph of 60 µM gLL-37_17-29_ (close to the MIC concentration of 53 µM), incubated with *M. luteus* for 4 h. All scale bars represent 500 nm.

**Fig. 3:**
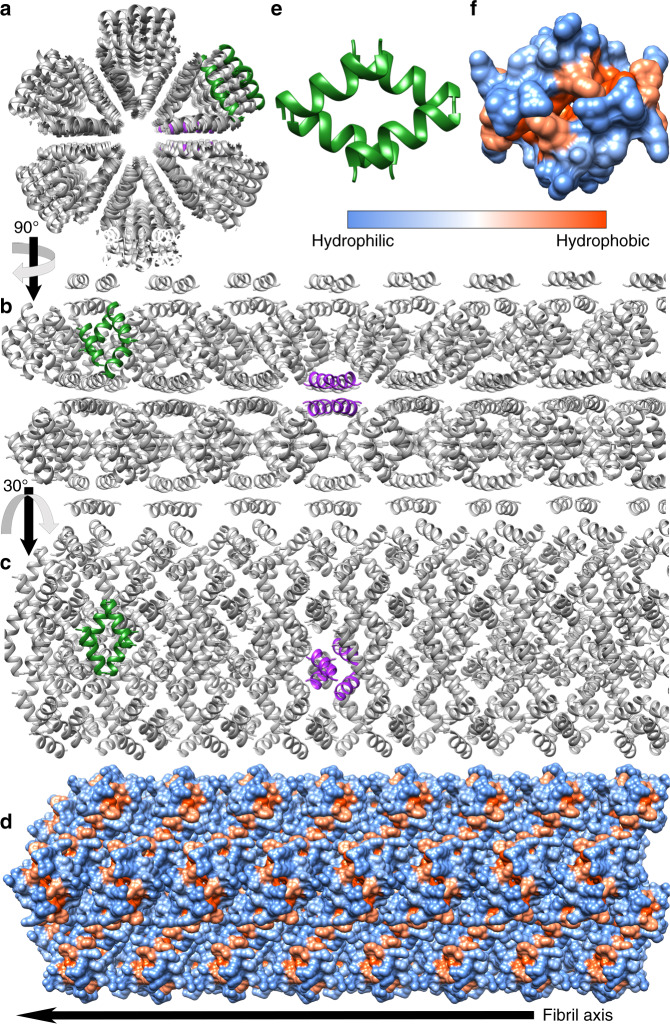
The crystal structure of hLL-37_17-29_. The crystal structure of hLL-37_17-29_ was determined at 1.35 Å resolution. The crystal packing shows self-assembly of amphipathic helices into a densely packed, elongated hexameric fibril with a central pore. The fibril is composed of four-helix bundles with a hydrophobic core that associated via a network of polar interaction (Fig. 5). **a** The assembly is shown as grey ribbons, with two representative four-helix bundles colored green and purple to emphasize orientation in the fibril. **b** The view is rotated by 90˚ in relation to panel a, showing the structure along the fibril axis. **c** The view is rotated by 30˚ along the fibril axis compared to **b**. **d** The fibril, in the same orientation as in panel c, is shown in a surface representation, colored by hydrophobicity, according to the scale bar. **e** An isolated four-helix bundle shown as green ribbons. **f** The four-helix bundle, in the same orientation as in **e**, shown in a surface representation colored by hydrophobicity, according to the scale bar.

We found that hLL-37_17–29_ formed long (several micrometers and longer), ribbon-like, fibrils, visualized using transmission electron microscopy (TEM) (Fig. 2). Cryogenic electron microscopy (CryoEM) showed that the wide (few hundred nanometers) fibrils are composed of lateral association of thinner fibrils (Supplementary Fig. 4). The wide fibrils also formed in the presence, and interacted with *M. luteus* cells (Fig. 2). Our determination of the crystal structure of hLL-37_17–29_ at 1.35 Å resolution, revealed self-assembly of amphipathic helices into a densely packed and elongated hexameric structure forming a central pore (Table 1, Fig. 3 and Supplementary Movie 1). There were two helices in the asymmetric unit of the crystal, with 67% of their individual solvent accessible surface areas buried within the assembly, indicating compact packing. For comparison, in the PSMα3 cross-α structure, each helix is 62% buried in the fibril^22^. In contrast, structures of full-length LL-37, co-crystallized alone or with different lipids, resulted in different levels of assembly, including monomeric, dimeric, tetramers and fiber-like structure of oligomers^44^. The latter was observed when full-length LL-37 was co-crystallized with dodecyl phosphocholine (PDB ID 5NNT), showing a repetitive architecture of juxtaposed “head-to-tail” amphipathic helices, with interactions mediated by detergent molecules^44^. This structure formed a much looser packing compared to the LL-37_17–29_ structure, with only 45% of the helix buried within the protein assembly. Overall, the generation of the hLL-37_17–29_ active core allows antibacterial activity along with the formation of highly stable fibrils.

**Table 1:** Data collection and refinement statistics (molecular replacement).

	Gorilla LL-37_17-29_	Human LL-37_17-29_
PDB accession code	6S6N	6S6M
Beamline	ESRF ID23-2	EMBL P14 PETRA III
Date	12 May 2018	7 July 2018
*Data collection*		
Space group	P 61 2 2	P 61 2 2
*Cell dimensions*		
*a*, *b*, *c* (Å)	41.15 41.15 57.28	41.45 41.45 57.47
α, β, γ (°)	90.0 90.0 120.0	90.0 90.0 120.0
Wavelength (Å)	0.873	0.976
Resolution (Å)	57.3–1.1 (1.13–1.10)	35.9–1.35 (1.39–1.35)
R-factor observed (%)	5.6 (55.2)	5.6 (78.8)
*R*_meas_ (%)^a^	5.7 (59.8)	6.0 (83.9)
*I* / sigma	31.1 (3.1)	21.5 (3.5)
Total reflections	222293 (5198)	59815 (4470)
Unique reflections	12186 (808)	6782 (505)
Completeness (%)	99.4 (92.1)	98.7 (99.8)
Redundancy	18.2 (6.4)	8.8 (8.9)
CC_1/2_ (%)^b^	99.9 (90.4)	99.7 (89.7)
*Refinement*		
Resolution (Å)	35.6–1.1 (1.13–1.10)	19.5–1.35 (1.39–1.35)
Completeness (%)	99.4 (92.1)	98.7 (99.8)
No. reflections^c^	10967 (727)	6103 (452)
*R*_work_ (%)^d^	16.0 (47.8)	23.2 (39.9)
*R*_free_ (%)	17.9 (55.9)	26.8 (39.2)
No. atoms	307	300
Protein	123 (Chain A)	122 (Chain A)
	128 (Chain B)	147 (Chain B)
Water	56	31
*B*-factors		
Protein	11.4 (Chain A)	17.2 (Chain A)
	10.5 (Chain B)	16.3 (Chain B)
Water	28.8	28.1
*R.m.s. deviations*		
Bond lengths (Å)	0.014	0.011
Bond angles (°)	1.708	1.613
Clash score^80^	1.83	1.71
Molprobity score^80^	0.94	0.92
Molprobity percentile^80^	99th percentile	99th percentile
Number of xtals used for scaling	One crystals	One crystal

Values in parentheses are for highest-resolution shell.

^a^*R*_meas_ is a redundancy-independent R-factor defined in ref. ^81^.

^b^CC_1/2_ is percentage of correlation between intensities from random half-datasets^82^.

^c^Number of reflections corresponds to the working set.

^d^*R*_work_ corresponds to working set.

**Fig. 4:**
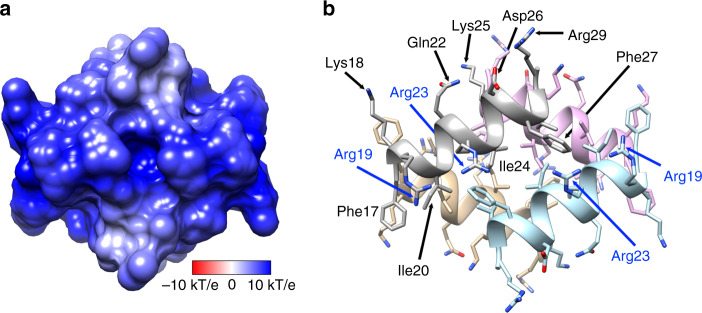
Positively charged electrostatic surface of the four-helix bundle of hLL-37_17-29_. **a** A projection of the electrostatic potential (φ) onto the molecular surface of the four-helix bundle of hLL-37_17-29_; the scale bar indicates φ ranges between −10 kT/e (dark red) and 10 kT/e (dark blue). **b** The bundle is displayed as ribbons, in the same orientation as in panel a, with side chains shown as sticks. The ribbons and carbons of each of the four helices are colored differently (gray, light blue, tan and pink) and non-carbon atoms are colored by atom type (oxygen in red and nitrogen in blue). Residues are labeled. Arg19 and Arg23 (labeled blue) from two helices lie across each side of the bundle.

**Fig. 5:**
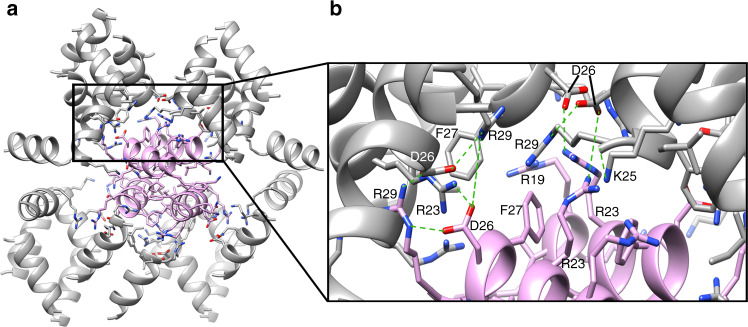
Interfaces of the four-helix bundle with surrounding helices in the fibril assembly. **a** One representative four-helix bundle is shown by pink ribbons, and surrounding helices are colored grey. Side chains of the “pink” bundle and residues of surrounding helices which contact the bundle are shown as sticks, colored by atom type (oxygen in red and nitrogen in blue). The asymmetric unit of the crystal contains two chains which are almost identical (RMSD of 0.13 Å), thus, the bundle shows almost four identical interfaces. **b** A zoom-in view. Potential polar interactions (up to 4.3 Å in distance) are indicated by green dotted lines: Asp26 can form inter-helical electrostatic interactions with Arg23 and with Arg29 from different helices, and intra-helical electrostatic interactions with Arg29. In addition, Lys25 can form electrostatic interactions with the negatively charged C-terminus of an adjacent helix. Overall, each helix shows four inter-helical and one intra-helical electrostatic interactions. In addition, Phe27 faces the middle of the interface, contributing to hydrophobic packing.

The structure of LL-37_17–29_ lacked amyloid continuous sheets with individual molecules stacked perpendicular to the fibril axis, and correspondingly did not bind the amyloid indicator dye Thioflavin T, in contrast to the cross-α amyloid fibrils of PSMα3^22^ (Supplementary Fig. 5). Rather, the fibrillar assembly of LL-37_17–29_ was comprised of associating four-helix bundles, each stabilized by a closely packed hydrophobic core (Fig. 3e, f). Arginine residues are lined on the surface of the bundles, with side chains extending outwards, providing an overall highly positively charged molecular electrical potential surface (Fig. 4). The interfaces between bundles comprises a network of polar interactions, including potential salt bridges between Asp26 and Arg23/Arg29 from two adjacent helices, and between Lys25 and the C-terminus of an adjacent helix (Fig. 5). Due to the symmetry in the structure, each bundle of four helices could form 16 inter-helical polar interactions with adjacent helices in the assembly (Fig. 5). In addition, Asp26 could potentially form a salt bridge with Arg29 on the same helix. Such intra-helical salt-bridges are associated with increased α-helical stability^45^. Phe27, facing towards another Phe27 residue from an adjacent helix, contributes to hydrophobic packing between bundles.

### LL37_17–29_ fibrils are stable

**Fig. 6:**
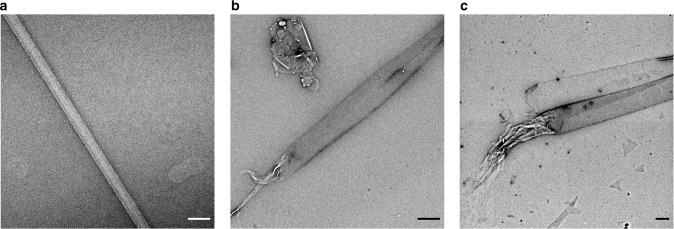
Thermostability of hLL-37_17-29_ fibrils. Transmission electron micrographs of 2 mM LL-37_17-29_ incubated for 3 days. **a** The sample was heated to 60 °C for 10 min. **b** The sample was incubated for additional 24 h at 37 °C after the 60 °C heat shock. **c** The sample was heated to 80 °C for 10 min All scale bars represent 500 nm.

The overall stable assembly of LL-37_17–29_, which includes a network of polar interactions and hydrophobic packing, corresponded with the thermostability of the fibrils, as visualized by electron micrographs after heating to 60 or 80 °C (Fig. 6). Some disassembly at the edge of the wide fibrils, into thinner fibrils, could be observed after the 80 °C heat shock, or after further incubation following the 60 °C heat shock exposing a lateral fibril association. In comparison, collagen fibrils, which provides physical support to tissues, disintegrate upon heating to 65 °C^46^. In addition, the surface charge and colloidal stability of the LL-37_17–29_ were assessed using zeta potential measurements^47^, showing concentration dependent values, reaching +25 mV at 1 mM (Supplementary Table 2). Congruently, other positively charged biological polymers were previously proposed for antimicrobials and drug delivery applications^48,49^.

### LL37_17–29_ fibrils interact with bacterial cells

The fibrillar assembly in the crystal structure created alternating hydrophobic and polar (positively charged) zigzagged belts on its surface (Fig. 3d), suggesting interactions with and disruption of negatively charged lipid bilayers, such as bacterial membranes^50^. Confocal microscopy images of fluorescein isothiocyanate (FITC)-labeled hLL-37_17–29_, which showed antibacterial activity similar to that of the unlabeled peptide (Supplementary Fig. 6), confirmed aggregation and co-localization of hLL-37_17–29_ with bacterial cells (Supplementary Fig. 7 and Supplementary Movie 2).

### Structure-guided mutagenesis supported functional fibrils

The role of the self-assembly in the antibacterial activity of hLL-37_17–29_ was examined using single-point mutations designed based on the solved crystal structure. Alanine substitution of Ile24, the most buried residue within the core of the four-helix bundle (Supplementary Fig. 8 and Supplementary Table 3), abolished antibacterial activity against *M. luteus* (Fig. 1) and against *S. hominis* (Supplementary Fig. 3). This mutation failed to exhibit peptide assembly around bacterial cells (Supplementary Fig. 9). Correspondingly, confocal microscopy images of the FITC-labeled I24A mutant, which, like the unlabeled peptide, was also ineffective against *M. luteus* (Supplementary Fig. 6), indicated absence of peptide aggregation (Supplementary Fig. 7). Likewise, the I24S, I24K, I24Q and I24D mutations all abolished antibacterial activity against *M. luteus* (Fig. 1), and confocal microscopy images of the FITC-labeled I24S inactive mutant (Supplementary Fig. 6) showed no detectable aggregation (Supplementary Fig. 7). Zeta potential measurements of the I24A inactive mutant showed significantly lower values compared to the native sequences, reaching only +14 mV at 1 mM (Supplementary Table 2). Since I24A is a conservative substitution with no change in net charge, we attribute the lower zeta potential values to lack of an organized particle assembly by the mutant.

Phe27 is another residue significantly buried within the fibrillar assembly (Supplementary Table 3), forming contacts with residues on the four-helix bundle and with other helices in the fibrillar assembly (Supplementary Fig. 8). The F27A mutation abolished antibacterial activity against *M. luteus* (Fig. 1) and failed to aggregate when incubated with the bacteria (Supplementary Fig. 9). In contrast to the buried Ile24 and Phe27, Gln22 was the least buried residue in the assembly, forming minimal contacts within adjacent helices (Supplementary Fig. 8 and Supplementary Table 3). Consequently, the Q22A mutation resulted in minimal change in activity against *M. luteus* (a MIC of 33 µM; Fig. 1) and was also active against *S. hominis*, with a MIC of 53 µM (Supplementary Fig. 3). Electron micrographs of the Q22A mutant showed large fibrous nanostructures contacting the bacterial cells (Supplementary Fig. 9). Correspondingly, confocal microscopy images of the FITC-labeled Q22A, which was slightly less active than the unlabeled Q22A peptide (Supplementary Fig. 6), formed aggregates which co-localized with the bacterial cells (Supplementary Fig. 7). Overall, the mutagenesis analyses indicated the importance of hLL-37_17–29_ self-assembly in its antibacterial activity and in direct interactions with bacterial cells, and supported a plausible active fibril arrangement.

Investigation of the N-terminal residues which are not buried within the assembly (Supplementary Table 3) but which face the central pore, showed that the F17A and K18A mutants display a similar reduction in antibacterial activity against *M. luteus*, with a MIC of 60 µM (Fig. 1). Maintaining the positive charge via a K18R substitution, showed a very similar MIC to that of hLL-37_17–29_, while the K18H substitution showed slightly reduced activity (Fig. 1). In contrast, substitution to the polar but uncharged glutamine (K18Q) fully abolished activity (Fig. 1). The results suggest that the two residues facing the pore are important for activity, with the positive charge of Lys18 being the critical determinant. We therefore cannot conclude about the specific role of the central pore in activity, as the effect of substitutions might be related to the reduced positive charge^51,52^ regardless of its structural location.

### Gorilla LL-37_17–29_ showed a similar fibril architecture

**Fig. 7:**
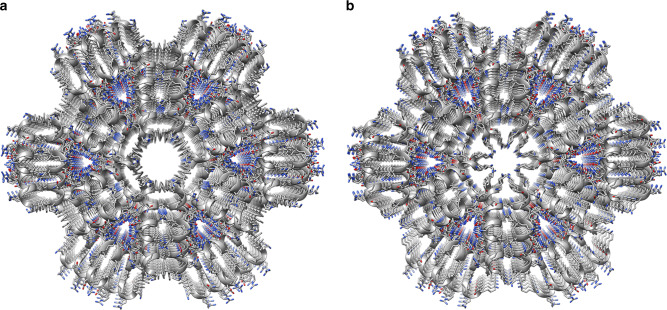
Human and gorilla LL-37_17-29_ share fibrillary architecture but differ in central pore properties. Comparison of human (**a**) and gorilla (**b**) LL-37_17-29_ crystal structures, shown in grey ribbons with side chain shown as sticks colored by atom type (oxygen in red and nitrogen in blue). The view is down the fibril axis, showing the hexametric arrangement and the central pore. The overall structure of the two is highly similar (RMSD of 0.15 Å for the asymmetric unit, comprising two helices and a similar space group and unit cell dimensions (Table 1)). The N-termini of the helices, with Phe17 in hLL-37_17-29_ or Ser17 in gLL-37_17-29_, and Lys18, line the central pore. The pore of gLL-37_17-29_ is more occluded, due to the orientation of the lysine residues extending into the pore. In the hLL-37_17-29_ structure, the lysine residues are almost perpendicular to the cross-section of the pore.

The gorilla LL-37 (gLL-37) sequence contains two amino acid substitutions when compared to hLL-37, with one at position 17, the first residue of LL-37_17-29_, substituting phenylalanine with serine (corresponding to a F17S mutation). gLL-37_17-29_ exhibited slightly weaker antibacterial activity against *M. luteus* compared to hLL-37_17-29_, with a MIC of 53 µM (Fig. 1), similar to the effect of the F17A mutant. The 1.1 Å resolution crystal structure of gLL-37_17-29_ displayed a similar assembly compared to hLL-37_17-29_ (Table 1 and Fig. 7), with almost identical backbone positions and root-mean-square deviation (RMSD) of 0.15 Å, differing only in the two N-terminal positions that lined the central pore (Supplementary Fig. 10). Accordingly, both hLL-37_17-29_ and gLL-37_17-29_ formed wide fibrillary structures, as observed by cryogenic electron micrographs (Supplementary Fig. 4), and engaged in direct contact with *M. luteus* cells (Fig. 2).

## Discussion

To conclude, the atomic structures of human and primate antibacterial LL-37_17-29_ showed a functional supramolecular nanostructure of densely packed amphipathic helices. This assembly into stable fibrils with a surface forming hydrophobic/charged zigzagged belts can be used as scaffolds for wide-ranging applications in bio and nanotechnology, regenerative medicine and bioengineering^53^, with the invaluable advantage of an inherent antibacterial activity. Links between fibril formation and antimicrobial activity are accumulating^11–14,17–21,54^, and here, we provide atomic-level insight for such example. Further elucidation of the interplay between antimicrobial activity and fibril formation and morphology will aid the design of AMPs with enhanced potency, selectivity, stability, bioavailability, and shelf-life. Successful design of such functional nanostructures with tunable self-assembly might provide novel antibacterial therapeutics or coating of medical devices, and will may target other roles of AMPs in immunomodulation and in killing cancerous cells^9,10,42^.

The LL-37_17-29_ structure differs from known helical fibrils such as the toxic cross-α amyloid fibrils of PSMα3, and from structural fibrils, such as collagen, actin, and fibrinogen. It overall presents a type of self-assembly which, to the best of our knowledge, is distinct from other protein fibrils, with a role in direct killing of bacterial cells still to be fully determined. Despite the different arrangement, the sequence similarity between the human LL-37 and the bacterial PSMα3, and their shared ability to form helical functional fibrils, suggest a possible molecular or structural mimicry mechanism used by the bacteria to provide immune-evasive and survival strategies^55,56^. This also points to potential functional building blocks across kingdoms of life in the form of densely packed amphipathic helical fibrils, complementing the exciting hypotheses about short amyloid peptides serving as prebiotic information-coding molecules^57–59^.

## Methods

### Peptides and reagents

LL37_17-29_ sequences are derivatives of the human and gorilla cathelicidin antimicrobial peptides CAMP (UniProt IDs P49913 and Q1KLY3, respectively). hLL37_17-29_, gLL37_17-29_, their mutants, fluorescein isothiocyanate (FITC)-labeled peptides, and *Staphylococcus aureus* PSMα3 (UniProt ID H9BRQ7) were purchased from GL Biochem (Shanghai) Ltd. as lyophilized peptides, at >98% purity. Thioflavin T (ThT) was purchased from Sigma-Aldrich. Ultra-pure double distilled water (UPddw) were purchased from Biological Industries. Additional reagents and consumable are mentioned below. Peptide sequences are shown in Supplementary Table 4.

### Bacterial strains and culture media

*Micrococcus luteus* (*M. luteus*, an environmental isolate) was a kind gift from Prof. Charles Greenblatt from the Hebrew University of Jerusalem, Israel. An inoculum was grown in Luria-Bertani medium (LB), at 30 °C, 220 rpm shaking, 16 h^27^. *Staphylococcus hominis* (subsp. *hominis Kloos and Schleifer S. hominis)* was purchased from ATCC (ATCC® 27844™). An inoculum was grown in brain-heart infusion media (BHI), at 37 °C, 220 rpm shaking, 16 h.

### Determination of minimal inhibitory concentrations

*M. luteus* and *S. hominis inoculums were* diluted to an OD_600_ = 0.1. For the MIC experiments, LL37_17-29_ and mutants were dissolved in PBS, and FITC-labeled peptides were dissolved in UPddw. The peptide stock solutions were then diluted into the bacterial media. Control and blank samples contained everything but peptides or everything but bacteria, respectively. Experiments were performed in a sterile 96-well plate and final reaction volume was 100 µl. Bacterial growth (OD_595_) was measured during a 24 h incubation, at 30 °C, with 220 rpm shaking, by a plate reader (FLUOstar omega or CLARIOstar, BMG LABTECH). Appropriate blanks were subtracted, and the area under the curve (AUC) was calculated^60^ from the resulting growth curves. MIC values were defined as the minimal concentration of the peptide which yielded less than 20% of the AUC of the control (bacteria with no added peptides). All experiments were performed in triplicates and were averaged. The entire experiment was repeated at least three times on different days, and the mean was calculated from the averaged triplicates of all biological repeats. Error bars represent standard errors of the mean. Two-tailed unpaired t-tests were performed to compare the mean MIC values of tested mutant peptides or derivatives to that of LL37_17-29_.

### Confocal microscopy imaging of bacteria–peptide interaction

*M. luteus* were grown for 16 h and diluted to an OD_600_ = 0.1. FITC-labeled peptides were dissolved in UPddw, sonicated for 3 min, and then added to the bacteria suspension to a final concentration of 30–150 µM (as indicated in the relevant figure); final reaction volume was 100 µl. Control samples contained everything but the peptide or everything but the bacterium. All samples were incubated, in the dark, at 30 °C, with shaking at 220 rpm, for 4 h. Thereafter, 1 ml paraformaldehyde 4% (w/v in PBS) was placed over the samples, for 15 min, at room temperature, in the dark. After fixation, samples were washed three times with fresh PBS, and then treated with Hoechst 33342 (10 mg/ml). All samples were applied to µ-Slides VI 0.4 slides (Ibidi, 80666). Confocal images were acquired using an inverted confocal laser-scanning microscope LSM 710 (Zeiss) equipped with a C-Apochromat 40× water immersion objective lens (NA 1.2) and a Definite Focus unit in an environmental chamber set at 37 °C. The laser wavelengths for excitation were 405 nm (Hoechst) and 488 nm (FITC). Brightfield images were collected from the 405 nm laser. Emission was collected sequentially at 410–497 nm for Hoechst and at 493–797 nm for FITC. The pinhole was set for 1 µm. Image processing was done with the Fiji software.

Time-lapse imaging: *M. luteus* bacteria from inoculum were diluted to OD_600_ = 0.1 in LB. Hoechst 33342 (10 mg/ml) was added to bacteria suspension and incubated in the dark, for 10 min, at room temperature. Just before imaging, FITC-LL37_17-29_ was added to the suspensions, to a final concentration of 30 µM. The samples were applied to µ-Slides VI 0.4 slides. Images with a digital resolution of 2048 × 2048 pixels and 16-bit depth were acquired every 10 min, at room temperature, using a Zeiss LSM 710 confocal microscope. Images were then used to construct a movie using Fiji software. A Gaussian filter (*σ* = 2.0) was applied to the FITC channel for noise removal and brightness and contrast of the images were adjusted^61–63^.

### Thioflavin T fluorescence kinetic assay

Thioflavin T (ThT) powder was dissolved in UPddw to a stock solution of 2 mM, vortexed and filtered twice through a 0.22 µm syringe-driven filter unit. Lyophilized PSMα3 peptide was pre-treated as previously described^22^: PSMα3 was dissolved to 1 mg/ml in trifluoroacetic acid (TFA) and hexafluoroisopropanol (HFIP, 1:1 v/v), sonicated for 3 min in a sonication bath and left to air-dry in a chemical hood. Dried samples were stored at −20 °C. Just before the experiment, PSMα3 was dissolved to 1 mM in UPddw, sonicated for 3 min and immediately transferred to ice. Thereafter, it was diluted to 50 µM with reaction buffer (200 µM ThT, 10 mM sodium phosphate buffer, pH = 8, and sodium chloride 150 mM), centrifuged at 12,500 rpm, for 10 min, in a pre-chilled centrifuge (4 °C). Lyophilized LL37_17-29_ peptide was dissolved in an identical reaction buffer to 1 mM. Blank solutions contained everything but the peptides. The reaction was carried out in a Greiner Bio-One black 96-well flat-bottom plate, immediately covered with a silicone sealing film (ThermalSeal RTS), and incubated in a plate reader (CLARIOstar or FLUOstar omega, BMG LABTECH) at 25 °C, with 220 rpm shaking for 20 s before each measurement. ThT fluorescence (excitation:438 ± 20 nm; emission: 490 ± 20 nm), was collected for at least 72 h (only 24 h are presented). All measurements were performed in triplicates and the entire experiment was repeated on at least three different days. Readings of blank solutions were subtracted. Error bars represent standard errors of the means.

### Transmission electron microscopy

Lyophilized LL37_17-29_ was dissolved in UPddw to a concentration of 1–5 mM and incubated, at 37 °C, for several days, as indicated in the individual Figures. For imaging the peptides in the presence of bacterial cells, *M. luteus* was grown for 24 h in LB. Approximately 1.5 × 10^9^ bacteria cells were washed three times with 10 mM potassium phosphate buffer at pH = 7.4. Lyophilized peptides (LL37_17-29_ and its mutants) were dissolved in this same buffer and added to the bacterial pellets, which were re-suspended to a final peptide concentration of 30–150 µM (as detailed in the relevant figures). Samples were then incubated, at 30 °C, with 220 rpm shaking, for 4 h. For testing Fibril thermostability, lyophilized hLL37_17-29_ was dissolved to 2 mM in UPddw and incubated at 37 °C for 3 days. After incubation, samples were moved to a heat block, pre-warmed to 60 or 80 °C (as indicated in the relevant figure), for 10 min. TEM samples were fixed on the EM grid directly from the heat block. The sample heated to 60 °C was further incubated at 37 °C for 24 h and then fixed on the grid.

TEM grid preparation and visualization were performed as follows. Samples (4–5 µl) were applied directly onto glow-discharged (easiGlow; Pelco, Clovis, CA, USA, 15 mA current; negative charge; 25 s time) 400 mesh copper grids, with a grid hole size of 42 µm, stabilized with Formvar/carbon (Ted Pella, Inc.). Samples of peptide alone were allowed to adhere for 60 s, and samples with *M. luteus* were allowed to adhere for 45 s. Samples were than stained with 1% uranyl acetate solution (Electron Microscopy Science, 22400-1) for 60 s (peptide alone) or 30 s (peptide with *M.luteus*), before being blotted with Whatman filter paper. Specimens were examined with a FEI Tecnai T12 G2 electron microscope, at an accelerating voltage of 120 kV, or a FEI Tecnai G2 T20 electron microscope, at an accelerating voltage of 200 kV.

### Cryogenic electron microscopy

Lyophilized human and gorilla LL37_17-29_ were dissolved in UPddw to 1–5 mM and incubated at 37 °C for 3-10 days (as indicated in the relevant figures). Another sample was prepared from 2 mM hLL37_17-29_ dissolved in 2.7 mM sodium dodecyl sulfate (SDS) (diluted in UPddw from a 40% stock). Of note, 2.7 mM SDS is at sub critical micelle concentration (CMC). Within a temperature-controlled chamber at 100% relative humidity, 3 µl of each sample were applied to a perforated carbon film supported by an electron microscope grid, which were pre-discharged, as described above. After 3 s, the drop was blotted by Whatman filter paper and liquids were vitrified through rapid plunging of the grids into liquid ethane at its freezing point^64^. Specimens were examined under a FEI Talos 200 C high-resolution electron microscope, at an accelerating voltage of 200 kV, using a Gatan 626 cryo-holder. To minimize electron beam radiation damage, the low-dose imaging mode was used. Images were collected digitally by a FEI Falcon III direct-imaging camera and the TIA software, with the help of the “phase plates” (FEI), to enhance image contrast^65,66^.

### Zeta potential measurements

Lyophilized LL37_17-29_ and the I24A mutant were dissolved in UPddw and incubated at 0.01, 0.1, and 1 mM concentrations at 37 °C for 24 h. Electrophoretic mobility measurements were performed in 25 °C using a Malvern’s Zetasizer Ultra device while samples were measured in folded capillary zeta cell zeta cuvettes (Malvern, DTS1070). Zeta potential was deduced using the Smoluchowski approximation^47^. The cells and the electrodes were washed with ddw three times before and after each sample. Device was equilibrated for 120 s before each sample and then three consecutive measurements were performed. The presented data is the mean of three consecutive measurements and standard deviation indicates the route mean square of the three measurements.

### Crystallization conditions

Lyophilized human and gorilla LL37_17-29_ peptides were dissolved to 10 mM (17 mg/ml) in double distilled water, vortexed and centrifuged at 14,000 rpm, 4 °C, for 10 min. hLL37_17-29_ crystals were grown in a reservoir solution containing 0.2 M sodium acetate, 0.1 M Tris, pH 8.5, and 30% (w/v) polyethylene glycol 4000. gLL37_17-29_ crystals were grown in a reservoir solution containing 0.1 M HEPES, pH = 7.5, and 1.4 M sodium citrate. The crystals were grown at 20 °C, using the hanging-drop vapor diffusion technique. gLL37_17-29_ crystals were soaked in cryo-protectant solution which contained reservoir solution, 5% (v/v) 2-methyl-2,4-pentanediol (MPD) and 20% glycerol. Crystals were flash-frozen in liquid nitrogen before X-ray data collection.

### Structure determination and refinement

X-ray diffraction of gLL37_17-29_ was collected at the ID23-EH2 micro-focus beamline at the European Synchrotron Radiation Facility (ESRF), Grenoble, France. The wavelength of data collection was 0.873 Å. X-ray diffraction of hLL37_17-29_ was collected at the EMBL micro-focused beam P14, at the high brilliance 3rd Generation Synchrotron Radiation Source at DESY: PETRA III, Hamburg, Germany. The wavelength of data collection was 0.976 Å. Data indexing, integrating and scaling were performed using XDS and XSCALE^67^. Phases were obtained by molecular replacement using Phaser^68^. For the molecular replacement of gLL37_17-29_, a 13-residue poly-alanine idealized helix was used as the search model. For the molecular replacement of hLL37_17-29_, the structure of gLL37_17-29_ was used as the search model. Crystallographic refinements were performed with Refmac5^69^. Further model building was performed using Coot^70^ and illustrated with Chimera (including constructing Movie S1)^71^. The structures of human and gorilla LL37_17-29_ were determined at 1.35 Å and 1.1 Å resolution, respectively. In both structures, there were two peptide chains in the asymmetric unit and water molecules. There were no residues detected in the disallowed region at the Ramachandran plot. Crystallographic statistics are presented in Table 1.

### Sequence alignments and proteolytic digestion prediction

Sequence alignment between LL37_17-29_ and PSMα3 was performed using the MAFFT server^72^. Amino acids are colored by their physicochemical properties^73^. Proteolytic digestion sites in the human LL37 were predicted using the Expasy’s PeptideCutter Tool^74^.

### Calculations of structural properties

The electrostatic potential map, hydrophobicity and B factor scales presented in the figures were created using Chimera^71^. The values of the hydrophobicity scale were according to Kyte and Doolittle^75^. The electrostatic potential was calculated using APBS-PDB2PQR^76^. Helix amphipathicity and the hydrophobic moment (Supplementary Table 1) were calculated with HeliQuest^77^.

### Solvent-accessible surface area calculations

Solvent-accessible surface areas (SASAs) were calculated using AREAIMOL, with a probe radius of 1.4Å^78,79^, via the CCP4 package^69^. The solvent-accessible buried surface area of each chain in the asymmetric unit was calculated as the area difference between the isolated chain and the chain within the fibril assembly, and is presented as the percentage of the total SASA of the chain. The SASA per residue within different isolated helical assemblies are presented in Supplementary Table 3.

### Reporting summary

Further information on research design is available in the Nature Research Reporting Summary linked to this article.

## Supplementary information

Supplementary Information

Peer Review File

Description of Additional Supplementary Information

Supplementary Movie 1

Supplementary Movie 2

Reporting Summary

## Data Availability

Coordinates and structure factors for the X-ray crystal structures have been deposited in the protein data bank (PDB) with accession codes 6S6M (hLL37_17-29_) and 6S6N (gLL37_17-29_). Other data are available from the corresponding author upon reasonable request. Source data are provided with this paper.

## Source data

Source Data
