# Supplementary material for: The Human LL-37(17-29) antimicrobial peptide reveals a functional supramolecular structure
Source: Nat Commun. 2020 Aug 4;11:3894. doi: 10.1038/s41467-020-17736-x (PMC7403366; doi:10.1038/s41467-020-17736-x)
Supplement: Supplementary file 1 — Supplementary Information [file 41467_2020_17736_MOESM1_ESM.pdf]

Supplementary Information

The Human LL-37(17-29) Antimicrobial Peptide Reveals a Functional Supramolecular Structure

Yizhaq Engelberg and Meytal Landau

Supplementary Figure 1. Sequence alignment of human LL-37 and bacterial PSMα3

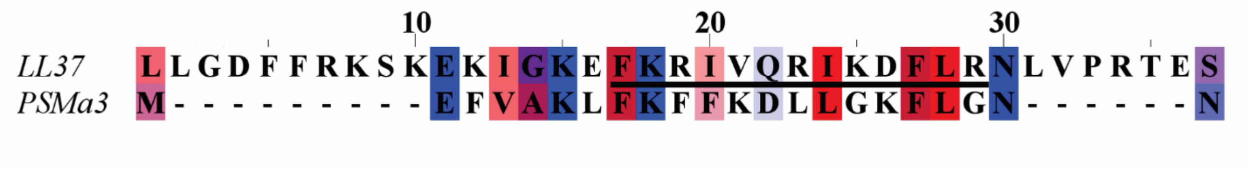

Sequence alignment between human LL-37 (UniProt ID P49913) and *S. aureus* PSMα3 (UniProt ID H9BRQ7). Amino acids are color-coded by their physicochemical properties[1]. Identity and similarity between the two sequences were 19% and 24%, respectively. The hLL-$37_{17\text{-}29}$ segment within the full sequence of hLL-37 is underlined and constitutes the most conserved region between the two peptides, with 31% identity and 39% similarity to the equivalent segment in PSMα3.

Supplementary Figure 2. hLL-37$_{17-29}$ concentration-dependent inhibition of *M. luteus* growth

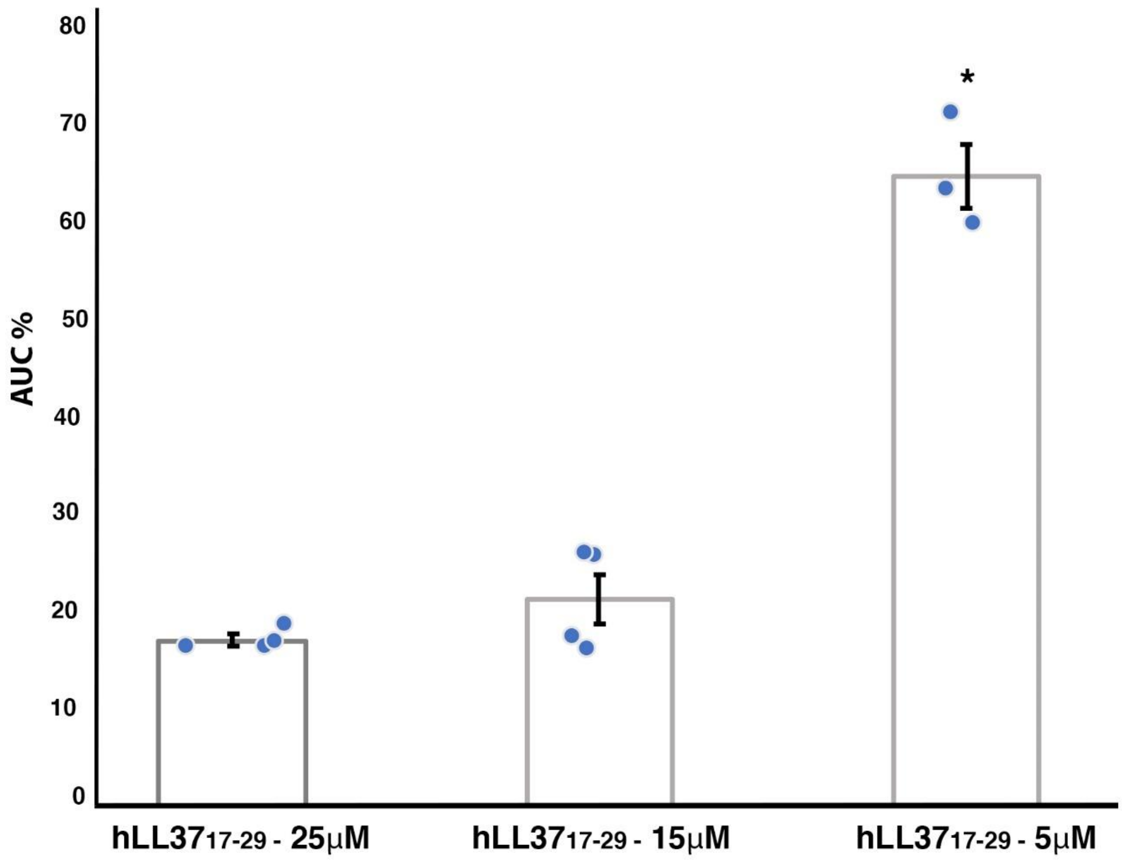

The integrated area under the curve (AUC) of bacterial growth over 24h is presented as percentage of control (peptide-free samples). hLL-37$_{17-29}$ at concentrations of 25, 15 and 5 μM present AUC percentage values of 17, 22 and 66%, respectively. All experiments were performed in triplicates, which were averaged. The experiments were performed at least three times, on different days. Dots represent the measured value for each individual experiment. Error bars represent the standard deviation of the mean (from the averaged triplicates of all biological repeats) and divided by the root of the number of repeats. For significance, a paired two-sample t-test, assuming equal variances, was performed; *indicates $p<5x10^{-05}$ compared to hLL-37$_{17-29}$ 25μM. Source data are provided as a Source Data file.

Supplementary Figure 3. Inhibition of *S. hominis* growth by different hLL-37$_{17\text{-}29}$ mutants and FITC-conjugates

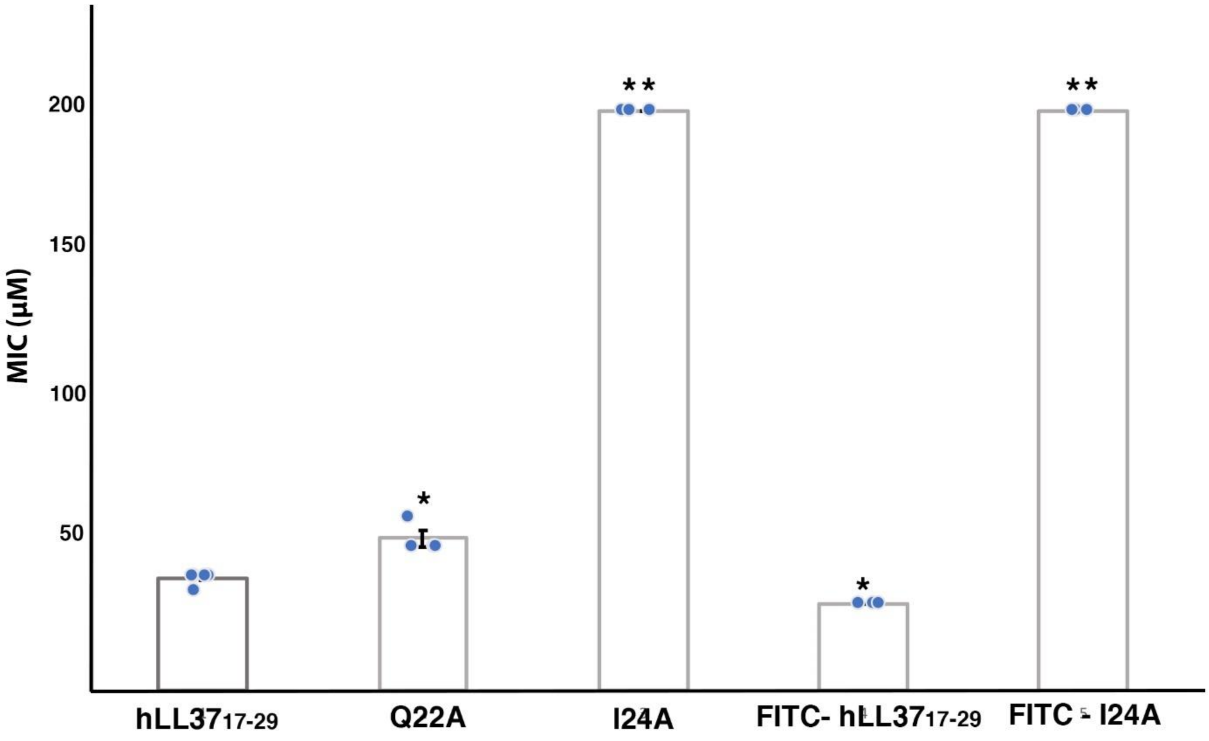

Growth inhibition of *S. hominis* by different hLL-37$_{17\text{-}29}$ mutants and FITC-conjugates, as expressed by the MIC values indicated above the bars. The highest tested concentration of the peptides was 200 µM. hLL-37$_{17\text{-}29}$ presented a MIC value of 39 µM. The active Q22A and inactive I24A mutants presented MIC values of 50 µM and >200 µM, respectively. The FITC conjugated of hLL-37$_{17\text{-}29}$ and the I24A mutant presented MIC values of 30 µM and >200 µM, respectively. All experiments were performed in triplicates, which were averaged. The experiments were performed at least three times, on different days. Dots represent the measured value for each individual experiment. Error bars represent the standard deviation of the mean (from the averaged triplicates of all biological repeats). A paired, two-sample Student's t-test, assuming equal variances, was performed; * indicates $p<0.001$, ** indicates $p<5\times10^{-10}$ compared to hLL-37$_{17\text{-}29}$. Source data are provided as a Source Data file.

Supplementary Figure 4. Cryo-electron micrographs of human and gorilla LL-37$_{17-29}$

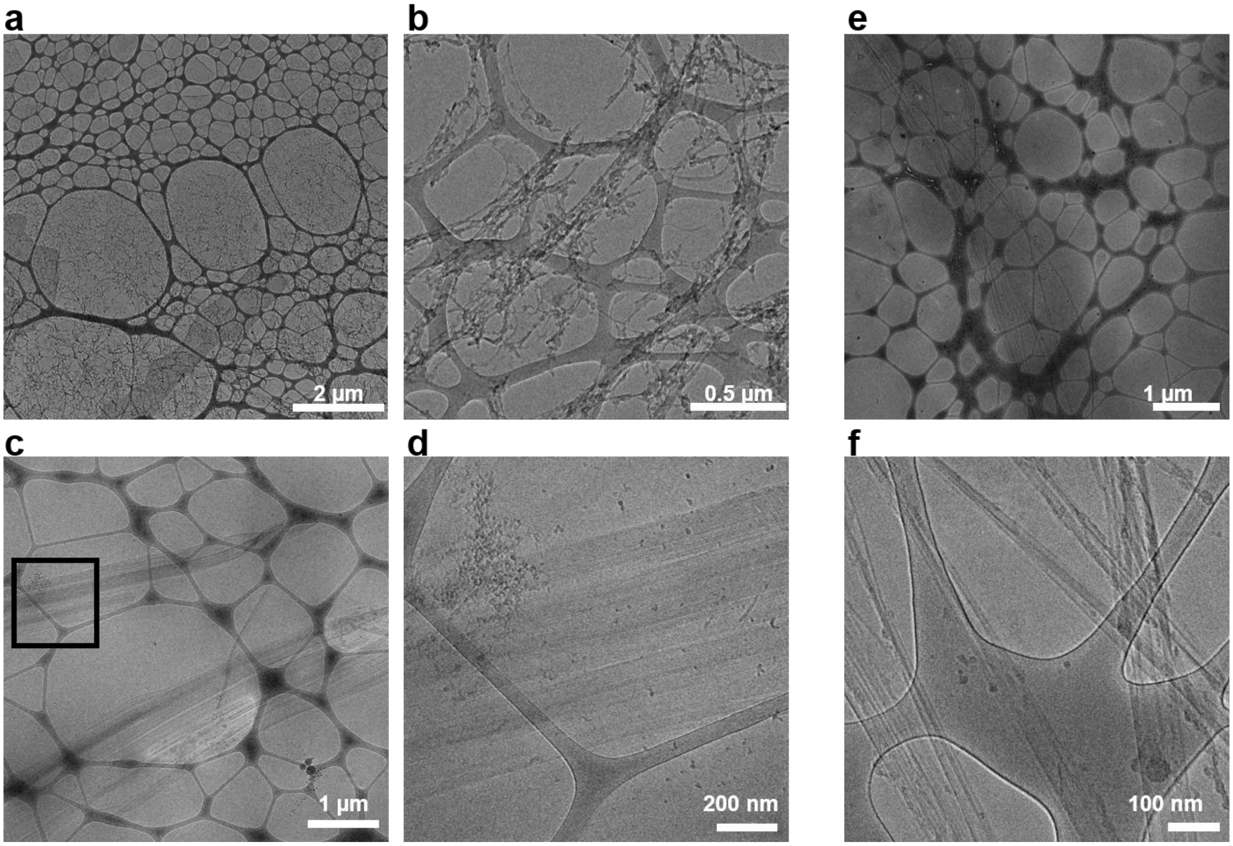

Cryogenic electron micrographs of human (**a-d**) and gorilla (**e-f**) LL-37$_{17-29}$. (**a-b**) Micrographs at two different magnifications show massive fibrillation of 5 mM hLL-37$_{17-29}$, after incubation at 37 ˚C, for three days. (**c-d**) Micrographs of 2 mM hLL-37$_{17-29}$ incubated with 2.7 mM SDS for 10 days, at 37 ˚C. The image in panel d is a zoom-in view of the boxed image in panel c. The micrographs display the formation of straight fibrils that bundle to form several hundred nanometer-wide ribbons, similar to what was observed in the negative-staining TEM images (Fig. 2). (**e-f**) Micrographs of 1 mM gLL-37$_{17-29,}$ incubated for three days, at 37 ˚C, show the formation of very long (several micrometers) and straight fibrils that also bundle into wide ribbon-like fibrils.

Supplementary Figure 5. ThT fluorescence kinetics of hLL-37$_{17-29}$ and PSMα3

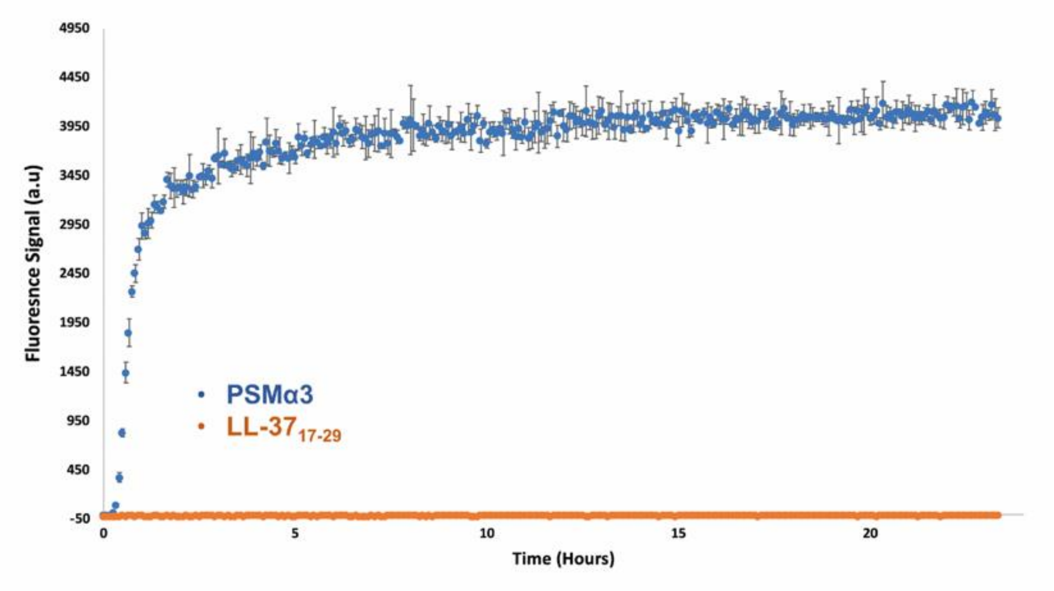

ThT fluorescence kinetics in the presence of 1 mM hLL-37$_{17-29}$ (orange curve) or 50 µM PSMα3 (blue curve). PSMα3 showed ThT binding, indicating rapid fibril formation, while hLL-37$_{17-29}$ failed to bind ThT. Measurements were performed in triplicates and values were averaged, appropriate blanks were subtracted, and the resulting values were plotted against time. Error bars represent standard errors of the mean. The entire experiment was repeated at least three times, on different days, showing similar results.

Supplementary Figure 6. Inhibition of *M. luteus* growth by FITC-labeled LL-37$_{17\text{-}29}$ derivatives

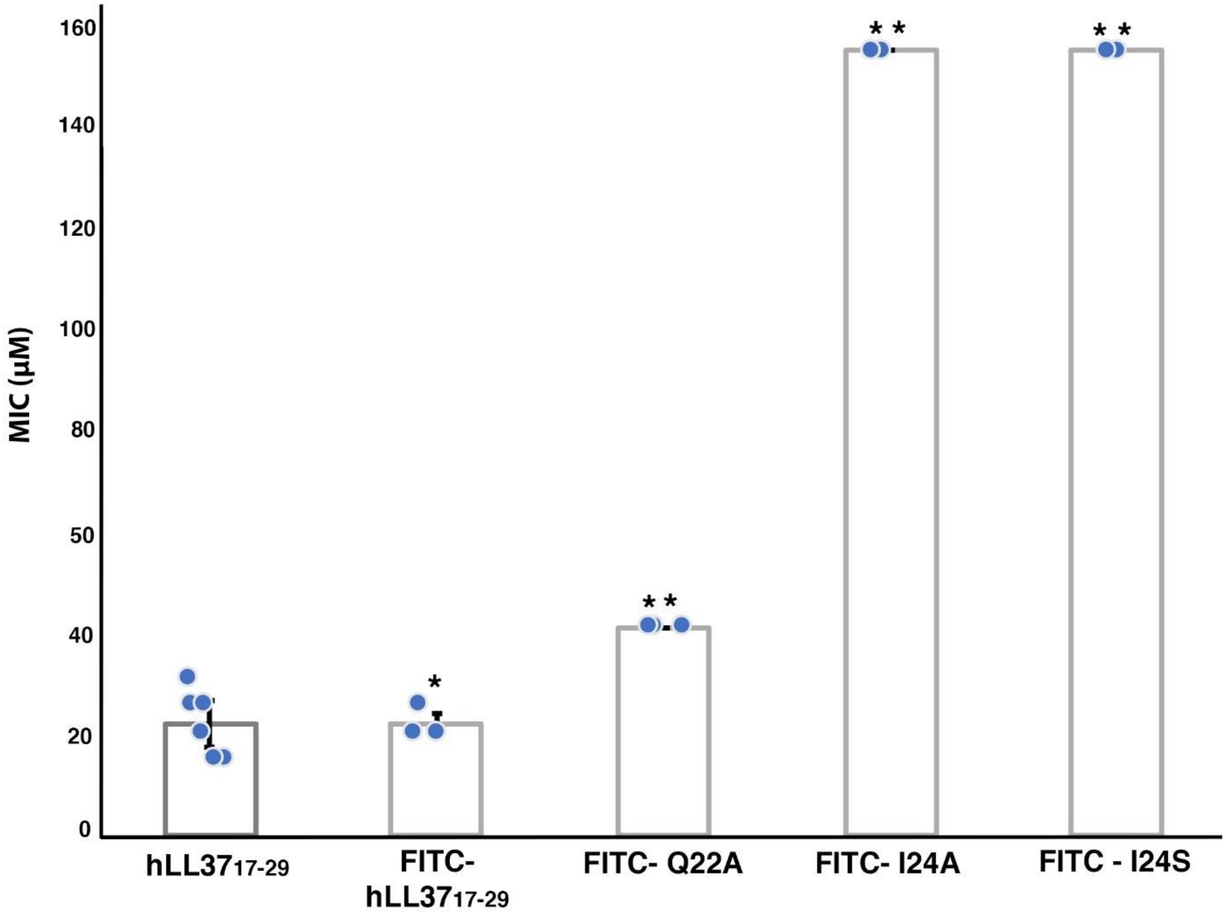

Growth inhibition of *M. luteus* by FITC-hLL-37$_{17\text{-}29}$ and its mutants is displayed as mean MIC values. The highest tested concentration of the peptides was 150 µM. hLL-37$_{17\text{-}29}$ and its FITC conjugate both showed the same mean MIC value of 22 µM. FITC conjugated of the Q22A mutant showed a MIC value of 40 µM. FITC conjugated of the I24A and I24S mutants showed a MIC value of >150 µM. All experiments were performed in triplicates, which were averaged. The experiments were performed at least three times, on different days. Dots represent the measured value for each individual experiment. Error bars represent the standard deviation of the mean (from the averaged triplicates of all biological repeats). A paired, two-sample Student's t-test assuming equal variances was performed; * indicates $p < 5x10^{-03}$ and ** indicates $p < 5x10^{-07}$ compared to hLL-37$_{17\text{-}29}$. Source data are provided as a Source Data file.

Supplementary Figure 7. Confocal microscopy images of *M. luteus* incubated with FITC-LL-37$_{17-29}$ and its mutants

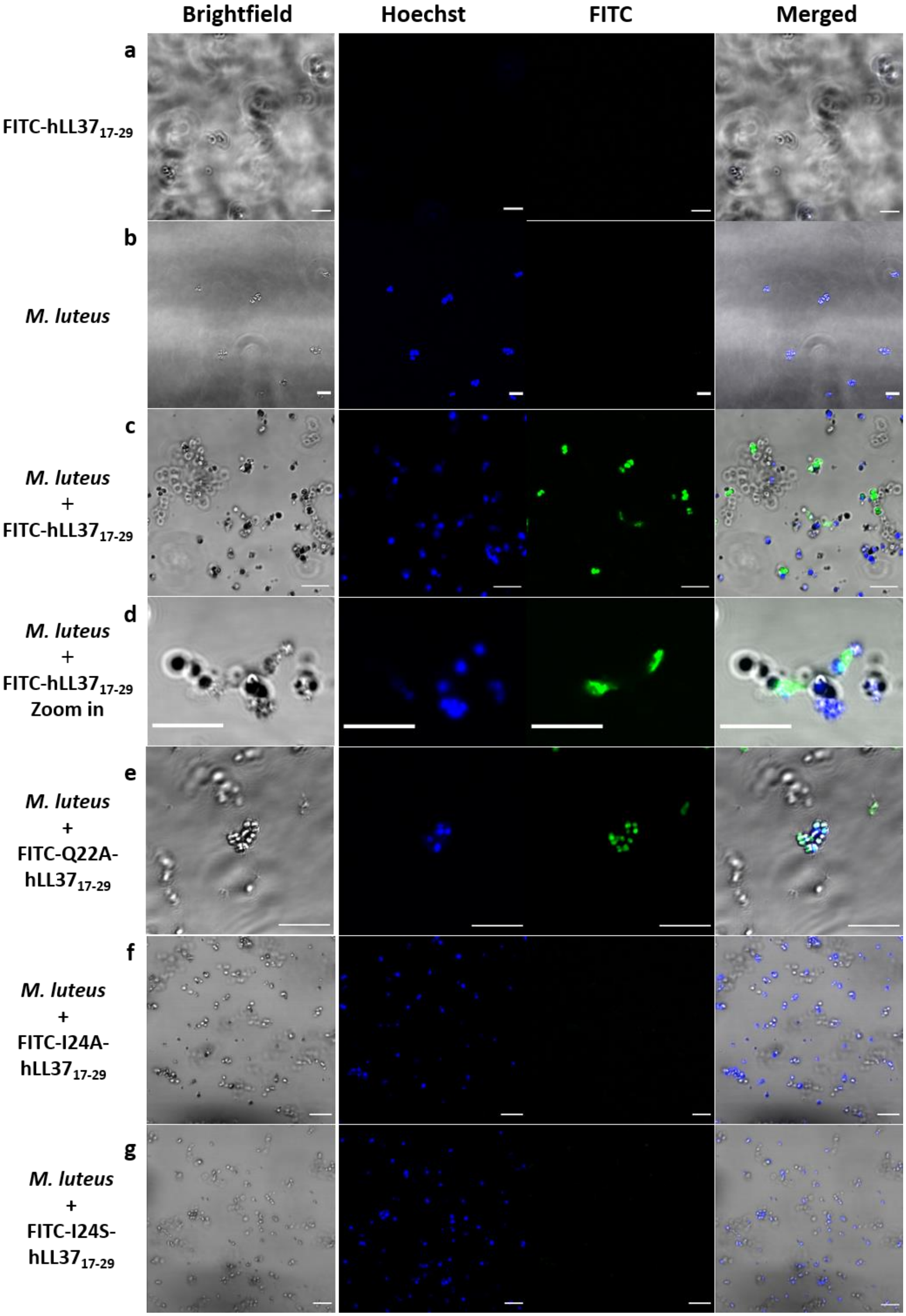

Representative confocal microscopy images of *M. luteus* incubated for 4 h with FITC-labeled LL-$37_{17\text{-}29}$ or mutants. The FITC channel was merged with the bright-field channel to show peptide location with respect to the bacteria (right column). A 20 µm scale bar is shown for all images. (**a**) A control sample containing 200 µM FITC-hLL-$37_{17\text{-}29}$ with no bacterial cells, showing no visible fluorescent signals. (**b**) A control sample containing *M. luteus* with no peptide, showing the blue stained bacterial cells. (**c-f**) *M. luteus* with: (**c-d**) 30 µM FITC-hLL-$37_{17\text{-}29}$ and a zoom-in view in panel d, (**e**) 50 µM FITC-hLL-$37_{17\text{-}29}$ Q22A, (**f**) 150 µM FITC-hLL-$37_{17\text{-}29}$ I24A or (**g**)150 µM FITC-hLL-$37_{17\text{-}29}$ I24S. The FITC-hLL-$37_{17\text{-}29}$ (**c-d**) and FITC-hLL-$37_{17\text{-}29}$ Q22A (**e**) aggregated (bright green foci) and co-localized with the bacterial cells. The FITC-hLL-$37_{17\text{-}29}$ I24A (**f**) and I24S (**g**) inactive mutants (Fig. 1) do not undergo aggregation.

Supplementary Figure 8. Structural location of mutated LL-37$_{17\text{-}29}$ residues

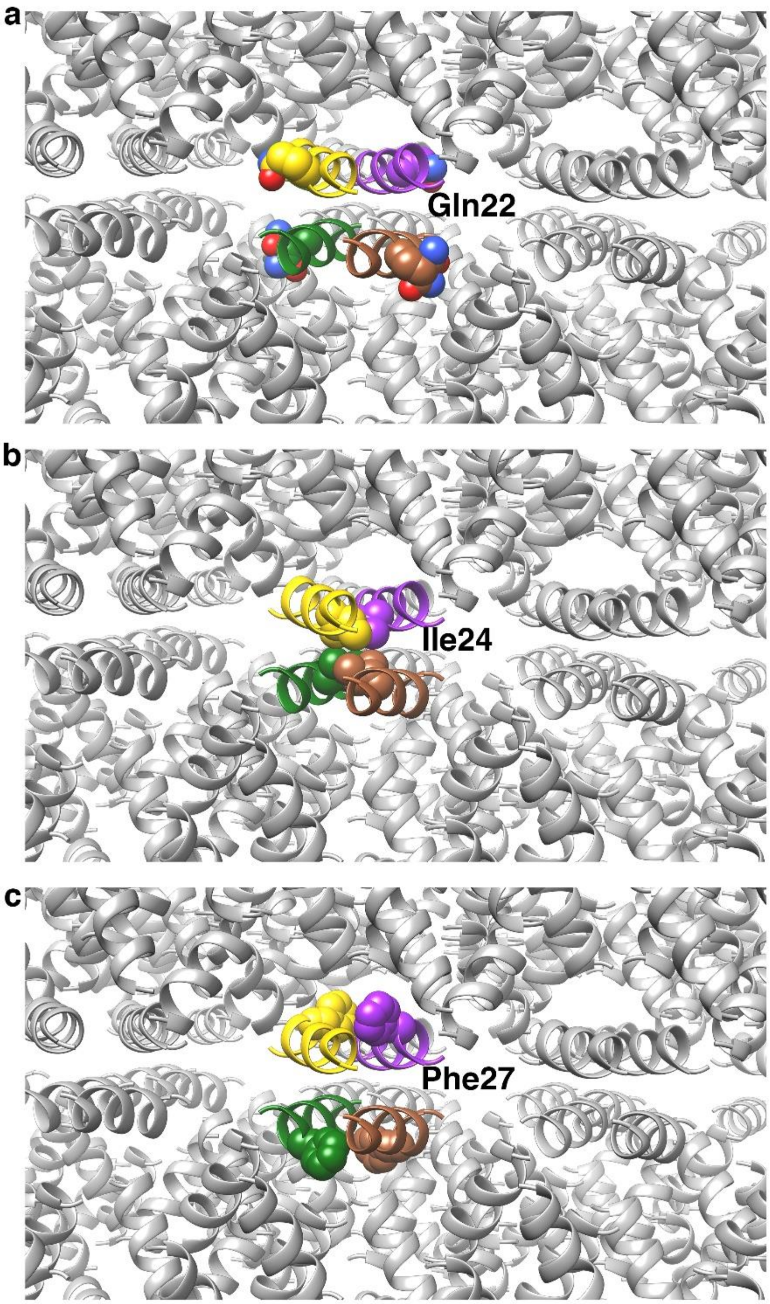

A zoom-in view of the fibrillar assembly, focusing on one four-helix bundle, with each helix colored differently. In each panel, residues that were substituted in our assays are individually shown using space-filling model. (**a**) Gln22 faces outward from the bundle, forming very few contacts with adjacent helices, with only 13% of its SASA buried in the assembly (Supplementary Table 3). (**b**) Ile24 is completely buried (95% of its SASA) inside the four-helix bundle. (**c**) Phe27 faces away from the bundle yet contacts both other residues on the same bundle and adjacent helices, with 85% of its SASA buried in the assembly.

Supplementary Figure 9. Electron micrographs of *M. luteus* incubated with hLL-37$_{17-29}$ mutants

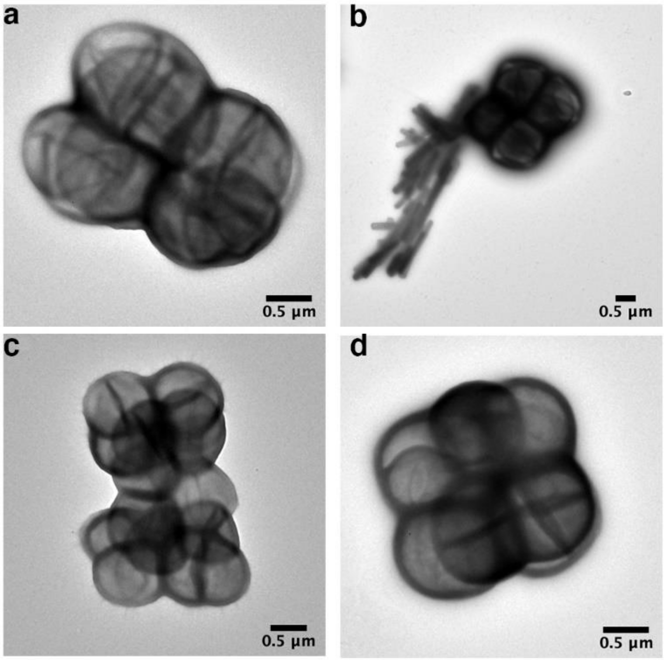

Transmission electron micrographs of *M. luteus* incubated with hLL-37$_{17-29}$ mutants. (**a**) A control sample containing *M. luteus* with no peptide added. (**b**) 50 µM hLL-37$_{17-29}$ Q22A active mutant incubated with the *M. luteus* displayed nano-fiber-like assemblies around and contacting the bacterial cells. The hLL-37$_{17-29}$ I24A (**c**) and F27A (**d**) inactive mutants, incubated at 100 µM with the bacteria, did not show apparent self-assembly.

Supplementary Figure 10. Structural alignment between human and gorilla LL-37$_{17-29}$ and residue flexibility

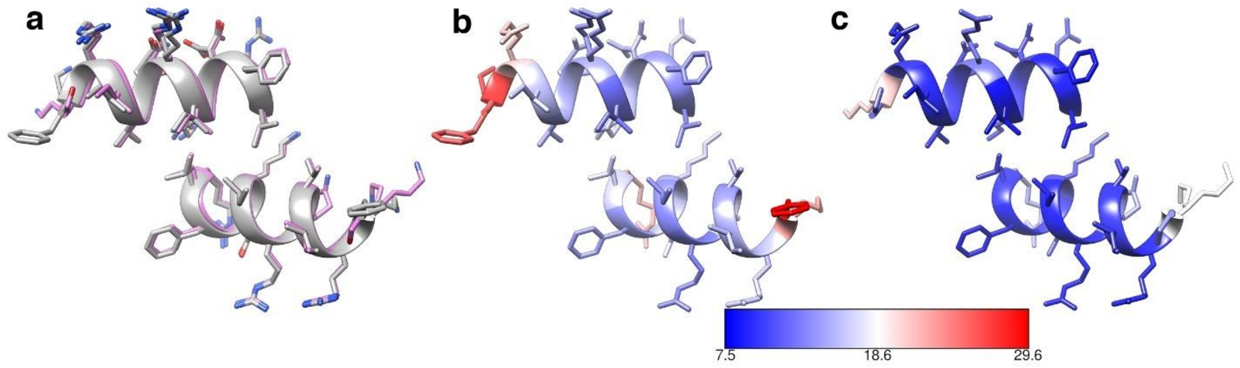

(**a**) Structural superimposition of the asymmetric unit, comprising two helices, of the human (grey) and gorilla (pink) LL-37$_{17-29}$ crystal structures, revealing a very similar structure with RMSD of 0.15 Å. (**b-c**) Human (**b**) and gorilla (**c**) LL-37$_{17-29}$ in the same orientation as in panel a, colored by average B (temperature) factors per residue, according to the scale bar, with blue-to-red indicating stable-to-flexible (ordered-to-disordered).

Supplementary Table 1. Properties of the amphipathic helices

	Hydrophobic moment	Hydrophobicity	Net charge
**hLL-37**	0.52	0.20	+6
**hLL-37$_{17-29}$**	0.85	0.31	+4
**gLL-37$_{17-29}$**	0.72	0.18	+4
**PSMα3**	0.56	0.54	+2

Supplementary Table 2. Zeta potential measurements of hLL37$_{17-29}$ and I24A

	0.01mM	0.1mM	1mM
**hLL37$_{17-29}$**	7.29 ± 1.43	21.5 ± 0.43	25.2 ± 0.47
**hLL37$_{17-29}$ I24A**	0.14 ± 0.67	7.79 ± 2.35	13.9 ± 0.45

Supplementary Table 3. Calculations of the solvent accessible surface area (SASA) per residue in the crystal structure of hLL-37$_{17-29}$

Residue on LL-37$_{17-29}$	Average percentage of area buried within the four-helix bundle[a]	Average percentage of area buried within the fibrillar assembly[b]	Average percentage of the residue area on the four-helix bundle buried by surrounding helices in the fibrillar assembly[c]	Percentage of area buried within the four-helix bundle versus the area buried in the fibrillar assembly[d]
Phe17	39%	57%	30%	27%
Lys18	24%	40%	21%	19%
Arg19	5%	58%	55%	4%
Ile20	59%	92%	81%	11%
Val21	87%	89%	21%	68%
Gln22	0%	13%	13%	0%
Arg23	21%	78%	73%	6%
Ile24	95%	96%	0%	96%
Lys25	46%	53%	13%	40%
Asp26	0%	71%	71%	0%
Phe27	59%	85%	64%	21%
Leu28	88%	98%	83%	15%
Arg29	7%	64%	61%	3%

[a] The number indicated is the percentage of the SASA per residue on an isolated helix versus the SASA of this residue within the four-helix bundle, averaged for the two chains. The higher the indicated percentage, the more buried the residue is on an isolated helix by surrounding helices within the four-helix bundle.

[b] The number indicated is the percentage of the SASA per residue on an isolated helix versus the SASA of this residue within the fibrillar assembly, averaged for the two chains. The higher the indicated percentage, the more buried the residue is on an isolated helix by surrounding helices within the fibrillar assembly.

[c] The number indicated is the percentage of the SASA per residue on the four-helix bundle buried by surrounding helices in the fibrillar assembly, averaged for the four chains. The higher the indicated percentage, the more buried the residue is by surrounding helices within the fibrillar assembly but not by other helices on the same four-helix bundle. This indicates the contact of each residue with helices surrounding the four-helix bundle.

[d] The percentage of SASA buried in the four-helix bundle versus the SASA buried in the fibrillar assembly. The higher the percentage difference per residue, the more contacts there are with surrounding residues on the four-helix bundle compared to residues on helices outside the bundle.

Supplementary Table 4. Peptide sequences

Peptide's Name	Sequence
LL37	LLGDFFRKSKEKIGKE**FKRIVQRIKDFLR**NLVPRTES
hLL37$_{17\text{-}29}$	FKRIVQRIKDFLR
gLL37$_{17\text{-}29}$ (F17S)	SKRIVQRIKDFLR
FITC-N- hLL37$_{17\text{-}29}$	FITC-FKRIVQRIKDFLR
hLL37$_{17\text{-}29}$ F17A	**A**KRIVQRIKDFLR
hLL37$_{17\text{-}29}$ K18R	F**R**RIVQRIKDFLR
hLL37$_{17\text{-}29}$ K18Q	F**Q**RIVQRIKDFLR
hLL37$_{17\text{-}29}$ K18H	F**H**RIVQRIKDFLR
hLL37$_{17\text{-}29}$ K18A	F**A**RIVQRIKDFLR
hLL37$_{17\text{-}29}$ Q22A	FKRIV**A**RIKDFLR
FITC- hLL37$_{17\text{-}29}$ Q22A	FITC- FKRIV**A**RIKDFLR
hLL37$_{17\text{-}29}$ I24A	FKRIVQR**A**KDFLR
FITC- hLL37$_{17\text{-}29}$ I24A	FITC- FKRIVQR**A**KDFLR
hLL37$_{17\text{-}29}$ I24S	FKRIVQR**S**KDFLR
FITC- hLL37$_{17\text{-}29}$ I24S	FITC- FKRIVQR**S**KDFLR
hLL37$_{17\text{-}29}$ I24K	FKRIVQR**K**KDFLR
hLL37$_{17\text{-}29}$ I24Q	FKRIVQR**Q**KDFLR
hLL37$_{17\text{-}29}$ I24D	FKRIVQR**D**KDFLR
hLL37$_{17\text{-}29}$ F27A	FKRIVQRIKD**A**LR
FITC- hLL37$_{17\text{-}29}$ F27A	FITC- FKRIVQRIKD**A**LR

Supplementary References

1. Livingstone, C. D. & Barton, G. J. Protein sequence alignments: a strategy for the hierarchical analysis of residue conservation. *Comput Appl Biosci* **9**, 745-756, (1993).
2. Mojsoska, B. *et al.* Peptoids successfully inhibit the growth of gram negative E. coli causing substantial membrane damage. *Sci Rep* **7**, 42332, (2017).
3. Yasir, M. *et al.* Comparative mode of action of the antimicrobial peptide melimine and its derivative Mel4 against Pseudomonas aeruginosa. *Scientific reports* **9**, 7063, (2019).
