# Supplementary material for: The Human LL-37(17-29) antimicrobial peptide reveals a functional supramolecular structure
Source: Nat Commun. 2020 Aug 4;11:3894. doi: 10.1038/s41467-020-17736-x (PMC7403366; doi:10.1038/s41467-020-17736-x)
Supplement: Supplementary file 3 — Description of Additional Supplementary Information [file 41467_2020_17736_MOESM3_ESM.pdf]

Description of Additional Supplementary Files

File Name: Supplementary Movie 1

Description: Crystal structure of hLL-3717-29 and the assembly of the peptide into fibril. The movie displays the hexameric crystal structure of hLL-3717-29 from different orientations and emphases. The helices are shown in grey ribbon presentation, with one representative four-helix bundle colored pink. The movie starts with a view into the fibril axis and zooms in to the representative bundle, showing its side chains. The orientation is then rotated 90˚ for a view along the fibril axis, and then further rotated along the fibril axis to display the overall helical assembly. The view is again zoomed into the bundle, showing side chains. The orientation is then rotated back to a view into the fibril axis.

File Name: Supplementary Movie 2

Description: Confocal microscopy imaging of live M. luteus incubated with FITC-hLL-3717-29. A series of confocal microscopy images was taken for about two hours, at intervals of 10 minutes, and then merged into a movie. The images reveal a rapid accumulation of FITC-hLL-3717-29 on the bacteria cells and the formation of peptide foci, indicating rapid aggregation in the presence of M. luteus. Depletion of the Hoechst blue signal was observed for bacterial cells localized with the peptide (green), indicating cell rupture and the release of DNA (Supplementary References 2&3). Bacterial cells with no accumulation of the peptide maintained a strong Hoechst blue signal.
