# Supplementary material for: The Human LL-37(17-29) antimicrobial peptide reveals a functional supramolecular structure
Source: Nat Commun. 2020 Aug 4;11:3894. doi: 10.1038/s41467-020-17736-x (PMC7403366; doi:10.1038/s41467-020-17736-x)
Supplement: Supplementary file 2 — Peer Review File [file 41467_2020_17736_MOESM2_ESM.pdf]

Editorial Note: This manuscript has been previously reviewed at another journal that is not operating a transparent peer review scheme. The manuscript was considered suitable for publication without further review at *Nature Communications*.
